# TRAIL-Mediated Suppression of T Cell Receptor Signaling Inhibits T Cell Activation and Inflammation in Experimental Autoimmune Encephalomyelitis

**DOI:** 10.3389/fimmu.2018.00015

**Published:** 2018-01-22

**Authors:** I-Tsu Chyuan, Hwei-Fang Tsai, Chien-Sheng Wu, Chi-Chang Sung, Ping-Ning Hsu

**Affiliations:** ^1^Department of Internal Medicine, Cathay General Hospital, Taipei, Taiwan; ^2^College of Medicine, Graduate Institute of Clinical Medicine, National Taiwan University, Taipei, Taiwan; ^3^College of Medicine, Fu Jen Catholic University, Taipei, Taiwan; ^4^College of Medicine, Graduate Institute of Clinical Medicine, Taipei Medical University, Taipei, Taiwan; ^5^Department of Internal Medicine, Taipei Medical University Shuang Ho Hospital, Taipei, Taiwan; ^6^Division of Rheumatology, Department of Internal Medicine, Far Eastern Memorial Hospital, Taipei, Taiwan; ^7^College of Medicine, Graduate Institute of Immunology, National Taiwan University, Taipei, Taiwan; ^8^Department of Internal Medicine, National Taiwan University Hospital, Taipei, Taiwan

**Keywords:** TRAIL, T cell receptor signaling, T cell activation, experimental autoimmune encephalomyelitis, apoptosis

## Abstract

**Objective:**

Tumor necrosis factor-related apoptosis-inducing ligand (TRAIL) induces cell apoptosis by transducing apoptosis signals after interacting with its receptor (TRAIL-R). Although the actual biological role of TRAIL remains to be elucidated, recent accumulating evidence implies that TRAIL regulates immune responses and immune cell homeostasis *via* an apoptosis-independent pathway, suggesting a novel immune-regulatory role of TRAIL in autoimmune diseases. The purpose of this study is to address the immune-regulatory role and molecular mechanism of TRAIL in regulating T cell activation in autoimmune diseases.

**Design:**

TRAIL was administered to mice to induce experimental autoimmune encephalomyelitis (EAE), and to evaluate its impact on neuroinflammation and disease activity. The effects of TRAIL on neuroantigen [myelin oligodendrocyte glycoprotein (MOG)_35–55_]-activated T cell proliferation and cytokine production were investigated. TRAIL-treated MOG_35–55_-activated splenic Th17 cells were further adoptively transferred into Rag1 KO mice to induce passive EAE. Gene expression profiles of CD4^+^ T cells from EAE mice treated with TRAIL were analyzed by RNA sequencing and transcriptome analysis.

**Results:**

TRAIL suppressed autoimmune encephalomyelitis and inhibited T cell reactivity to neuro-antigen in murine EAE, and the effects were dependent on TRAIL-R signaling. Moreover, TRAIL directly inhibited activation of MOG_35–55_-activated CD4^+^ T cells, resulting in suppression of neuroinflammation and reduced disease activity in adoptive transfer-induced EAE. Furthermore, TRAIL-R signaling inhibited phosphorylation of proximal T cell receptor (TCR)-associated tyrosine kinases in activated CD4^+^ T cells. Importantly, TRAIL/TRAIL-R interaction downregulated TCR downstream signaling genes in RNA sequencing and transcriptome analysis.

**Conclusion:**

TRAIL/TRAIL-R interaction regulates CD4^+^ T cell activation in autoimmune inflammation and directly suppresses T cell activation *via* inhibiting TCR signaling, suggesting that TRAIL-R serves as a novel immune checkpoint in T cell responses.

## Introduction

TRAIL is a type II transmembrane protein of the TNF superfamily, expressed in a wide range of tissues, and shares high homology with Fas ligand (1–4). TRAIL induces apoptosis *via* binding of its death-inducing receptors (5, 6). In humans, there are five TRAIL receptors including two death-inducing receptors [DR4/TRAIL-R1 (7) and DR5/TRAIL-R2 (3, 8)] and three decoy receptors [DcR1/TRAIL-R3 (3, 8), DcR2/TRAIL-R4 (9, 10), and osteoprotegerin (11)]. In mice, only one death-inducing receptor was identified that shares high homology with human DR5/TRAIL-R2 (mouse KILLER/DR5) (4). Although TRAIL induces apoptosis in many tumor cell lines, almost all primary cells are resistant to TRAIL-induced cell death (1, 2), and the actual biological role of TRAIL remains to be elucidated. Recent accumulating evidence implies an emerging role of TRAIL in modulating immune responses. TRAIL administration induced anti-inflammation in several autoimmune animal models (12–20). In mice with experimental autoimmune encephalomyelitis (EAE), TRAIL blockade (14) or TRAIL deficiency (21) increased neuroinflammation and enhanced disease activity, while inflammation was inhibited using genetically modified TRAIL-expressing cells (22) or TWEAK receptor-TRAIL fusion protein (23). In addition, recent studies (15–18) demonstrated that TRAIL suppressed joint inflammation and synovium-infiltrating lymphocytes in autoimmune arthritis models. Therefore, it is possible that TRAIL plays a critical role in regulating immune responses and maintaining immune cell homeostasis to prevent autoimmunity. However, the mechanism of TRAIL-mediated inhibition of inflammation and autoimmunity is still not clear.

TRAIL was implicated in regulating inflammation, mainly due to promoting apoptosis of lymphocytes and infiltrating immune cells. Nevertheless, recent accumulating evidence suggests that TRAIL inhibits autoimmune inflammation *via* an apoptosis-independent pathway (14, 15, 19). Moreover, TRAIL inhibits T cell receptor (TCR) signaling and suppresses T cell activation *in vitro* (24), and TRAIL suppresses inflammation by direct inhibiting T cell activation in inflammatory arthritis (18). All these results imply a novel immunoregulatory role of TRAIL in autoimmune diseases (18).

To further address the immune-regulatory role and molecular mechanism of TRAIL in regulating autoimmune diseases, in this study, we demonstrate herein that TRAIL suppresses neuroinflammation and inhibits T cell reactivity against neuroantigen in murine EAE, and the effects are dependent on TRAIL-R signaling. TRAIL-mediated suppression of TCR signaling directly inhibits T cell activation and thus reduces neuroinflammation. Our study indicates that TRAIL is a critical regulator of T cell activation in autoimmune inflammation and implies that TRAIL-R can serve as a novel immune checkpoint in T cell responses.

## Materials and Methods

### Animals

Wild-type (WT) C57BL/6 mice (female, 6–7 weeks old) and Rag1 knockout (Rag1 KO) mice (female, 6–7 weeks old) were housed under specific pathogen-free conditions and provided with standard food and water. TRAIL-R knockout (TRAIL-R KO) mice (C57BL/6 background, female, 6–7 weeks old) were obtained from Henning Walczak (UCL Cancer Institute, University College London, UK) (25). All animal work was conducted according to guidelines of the Association for Assessment and Accreditation of Laboratory Animal Care. All animal experiments were approved by the Animal Ethics Committee of the National Taiwan University Medical Center.

### Induction of EAE and Generation of Myelin Oligodendrocyte Glycoprotein (MOG)_35–55_-Activated Th17 Cells

Mice were immunized by a subcutaneous (s.c.) injection with an encephalitogenic cocktail (Hooke Laboratories, Lawrence, MA, USA) containing MOG_35–55_ (200 μg/mouse) and heat-killed *Mycobacterium tuberculosis* H37RA (500 μg/mouse) in complete Freund’s adjuvant (CFA). Pertussis toxin (250 ng/mouse, Hooke Laboratories) was intraperitoneally (i.p.) injected twice on the day of immunization and 24 h later. EAE symptoms (loss of mobility and limb paralysis) in mice were recorded daily from the day after immunization according with this scale: 0 = no symptoms; 1 = total loss of tail tonicity; 2 = hind limb weakness with difficulty righting; 3 = unsteady gait and one hind limb plegia; 4 = paraplegia with forelimb weakness; 5 = quadriplegia; and 6 = death.

For adoptive-transfer EAE experiments, donor mice were immunized as described above except without an injection of pertussis toxin. Twelve days after immunization, mice were sacrificed, and splenocytes (4 × 10^6^ cell/ml) were cultured with 50 µg/ml of MOG_35–55_ (Hooke Laboratories) under conditions favorable to the generation of Th17 cells: 8 ng/ml rmIL-23 (Biolegend, San Diego, CA, USA), 10 ng/ml rmIL-1α (Biolegend), 10 µg/mL anti-interferon (IFN)-γ (clone: XMG1.2) (Biolegend), and 10 µg/mL anti-interleukin (IL)-4 (clone: 11B11) (Biolegend) in RPMI 1640 medium (Life Technologies, Camarillo, CA, USA) supplemented with 10% fetal bovine serum (FBS; Thermo Scientific, Waltham, MA, USA) for 72 h. Cultured cells were harvested, and CD4^+^ T cells were purified using magnetic-activated cell sorting (MACS) beads (STEMCELL Technologies, Vancouver, Canada). MOG_35–55_-activated Th17 cells were then cultured in RPMI 1640 medium supplemented with 1% FBS for 24 h, followed by stimulation with anti-CD3 (1 µg/ml) and anti-CD28 (1 µg/ml) antibodies (Abs) in the presence or absence of TRAIL (10 µg/ml) for an additional 24 h and adoptively transferred into Rag1 KO mice (4 × 10^6^ CD4^+^ T cells/recipient) intravenously (i.v.). Recipient mice were monitored daily, and clinical scores were graded as described previously (26).

### Purification of TRAIL

Recombinant TRAIL proteins were purified as described previously (27) and used at 100 μg/mouse i.p. for EAE treatment. For self-purified TRAIL, in brief, the coding portion of the extracellular portion of the TRAIL molecule (amino acids 95–281) was subcloned into a pRSET(B)-His vector (Invitrogen, Groningen, the Netherlands) and expressed in an *Escherichia coli* expression system. The His-TRAIL fusion protein was purified by metal chelate column chromatography using Ni-NTA resin, according to the manufacturer’s recommendations (Qiagen, Hilden, Germany) and dialyzed. Lipopolysaccharide endotoxin was further removed from the purified TRAIL using an Acrodisc syringe filter (Pall, NY, USA) and reached the targeted endotoxin level of < 0.1 EU/ml as determined by a Pierce LAL Chromogenic Endotoxin Quantitation Kit (Thermo Scientific). For *in vitro* assays, TRAIL was purchased from Genescript (Piscataway, NJ, USA) used at a concentration of 10 µg/ml.

### Histological Analysis

On day 30 after EAE immunization, control and EAE mice treated with vehicle or the TRAIL, respectively, were sacrificed. The spinal cord from each mouse was fixed in 4% formalin for 12 h, embedded in paraffin, and cut into five pieces. Serial paraffin sections (5 µm) of the spinal cords were stained with hematoxylin and eosin and an anti-CD3 immunohistochemical Ab to assess tissue inflammation and T cell infiltration.

### Brain and Spinal Cord [Central Nervous System (CNS)] Mononuclear Cell Isolation

Brains and spinal cords (CNS) were isolated from experimental mice, passed through a nylon mesh, and digested in collagenase-VIII (1.4 mg/ml) and DNase-I (100 µg/ml) (Sigma-Aldrich, St. Louis, MO, USA) in Hank’s balanced salt solution medium for 1 h at 37°C with agitation. The digested supernatant containing CNS mononuclear cells was pelleted and further purified by centrifugation over a 30%/70% Percoll gradient (GE Healthcare, Wauwatosa, WI, USA). Purified CNS mononuclear cells were cultured with RPMI 1640 medium supplemented with 10% FBS.

### IL-17 and IFN-γ Enzyme-Linked Immunospot (ELISpot) Assay

Lymph node (LN) and CNS mononuclear cells were cultured and assayed for the frequency of antigen-specific IL-17- and IFN-γ-secreting cells using a dual-color ELISPOT kit (R&D Systems, Minneapolis, MN, USA). Briefly, 5 × 10^5^ LN and CNS mononuclear cells were cocultured with MOG_35–55_ (20 µg/ml) in 100 µl RPMI 1640 supplemented with 10% FBS and incubated for 24 h at 37°C. Spot-forming cells were revealed by (1) IL-17 (red): horseradish peroxidase (HRP)-conjugated detection Ab with AEC chromogen, and (2) IFN-γ (blue): biotin-conjugated detection Ab with alkaline phosphatase-conjugated streptavidin and BCIP/NBT chromogen. Spots were analyzed using the AID EliSpot Reader System (Autoimmun Diagnostika GmbH, Straßberg, Germany).

### Intracellular Cytokine Assay

Lymph node and CNS mononuclear cells were isolated and stimulated with phorbol 12-myristate 13-acetate (50 ng/ml) (Sigma-Aldrich) and ionomycin (1 µg/ml) (Sigma-Aldrich) for 5 h at 37°C in RPMI 1460 medium containing 10% FBS and GolgiSTOP (BD Biosciences, San Diego, CA, USA). IL-17 and IFN-γ production by LN and CNS CD4^+^ cells was measured with a mouse Th1/Th17 phenotyping kit (BD Biosciences). Briefly, stimulated cells were fixed with cold BD Cytofix™ buffer and permeabilized with BD Perm/Wash™ buffer, followed by staining with a BD Th1/Th17 phenotyping cocktail containing CD4 PerCP-Cy5.5 (clone: RM4-5), IL-17A PE (clone: TC11-18H10.1), and IFN-γ FITC (clone: XMG1.2) according to the manufacturer’s instructions. Samples were acquired using FACS Canto II flow cytometer (BD Biosciences), and the data were analyzed using Cell Quest software (Becton Dickinson, Franklin Lakes, NJ, USA).

### Cell Proliferation Assay

Proliferation was evaluated by measuring DNA synthesis as assessed by incorporation of tritiated [^3^H] thymidine. MOG_35–55_-activated splenic Th17 cells were synchronized for 24 h at 37°C with RPMI 1640 medium containing 1% fetal calf serum, followed by incubation at a density of 10^5^ cells/well in 96-well flat-bottomed plates precoated with medium, an anti-CD3 Ab (1 µg/ml), an anti-CD28 Ab (1 µg/ml), and the TRAIL (10 µg/ml), or a combination of anti-CD3/anti-CD28 Abs and the TRAIL for 96 h at 37°C, and each condition was tested in quadruplicate. [^3^H] Thymidine (1 μCi/well) was then added for an additional 16 h. Cells were lysed and transferred onto a UniFilter-96 GF/C (PerkinElmer, Waltham, MA, USA). [^3^H] Thymidine incorporated into DNA was quantified using a scintillation counter TopCount NXT (Packard, CT, USA). Results represent proliferation of cells with the indicated treatment and are expressed as the arithmetic mean of counts per minute (cpm) from quadruplicate analyses of indicated stimulated cultures.

### Enzyme-Linked Immunosorbent Assay (ELISA) for Cytokines

MOG_35–55_ -activated splenic Th17 cells were cultured in 96-well flat-bottomed plates (2 × 10^5^ cells/well) precoated with medium, an anti-CD3 Ab (1 µg/ml), an anti-CD28 Ab (1 µg/ml), and TRAIL (10 µg/ml), or a combination of anti-CD3/anti-CD28 Abs and TRAIL for 24 h at 37°C. Supernatants were collected, and levels of IL-2 (Biolegend) and IL-17 (Biolegend) were determined using ELISA kits according to the manufacturer’s protocol.

### Transcriptome Analyses

On day 15 after EAE induction, five control and EAE mice treated with either vehicle (200 µl/mouse/day, i.p.) or TRAIL (100 µg/mouse/day, i.p.) were pooled, and their spleens were harvested. CD4^+^ T cells were isolated from single-cell suspensions by MACS beads (STEMCELL Technologies). Total RNA was extracted with the Trizol^®^ Reagent (Invitrogen, Carlsbad, CA, USA) according to the instruction manual. Purified RNA was quantified at OD260 nm using an ND-1000 spectrophotometer (Nanodrop Technology, Wilmington, DE, USA) and qualitated using a Bioanalyzer 2100 (Agilent Technology, Santa Clara, CA, USA) with an RNA 6000 labchip kit (Agilent Technologies). For RNA library preparation and sequencing, all procedures were carried out according to the manufacture’s protocol from Illumina. Library construction of all samples used Agilent’s SureSelect Strand Specific RNA Library Preparation Kit for 75 single-end sequencing on the Solexa platform. The sequence was directly determined using sequencing-by-synthesis technology *via* a TruSeq SBS Kit. Raw sequences were obtained from the Illumina Pipeline software, bcl2fastq v2.0, which was expected to generate 20 × 10^6^ reads per sample.

Initially, the sequences generated were subjected to a filtering process to obtain qualified reads. Trimmomatics was implemented to trim or remove reads according to the quality score. Qualified reads after filtering low-quality data were analyzed using TopHat/Cufflinks for gene expression estimation. The gene expression level was calculated as fragments per kilobase of transcript per million mapped reads (FPKM). For the differential expression analysis, CummeRbund was employed to perform statistical analyses of gene expression profiles. The reference genome and gene annotations were retrieved from the Ensembl database. *p*-value of ≤0.01 were considered significant.

The data discussed in this publication have been deposited in NCBI’s Gene Expression Omnibus and are accessible through GEO Series accession number GSE108523 (https://www.ncbi.nlm.nih.gov/geo/query/acc.cgi?acc=GSE108523).

### Western Blot Analysis

Mouse CD4^+^ T cells from WT C57BL/6 or TRAIL-R-KO mice were enriched (STEMCELL Technologies) and stimulated at the indicated time point at 37°C with medium, an anti-CD3 Ab (1 µg/ml), an anti-CD28 Ab (1 µg/ml), and the TRAIL (10 µg/ml), or a combination of anti-CD3/anti-CD28 Abs and the TRAIL. Whole-cell protein was subsequently extracted using the PhosphoSafe Extraction Reagent (Merck Millipore, Darmstadt, Germany). Protein lysates were transferred onto a polyvinylidene difluoride membrane after sodium dodecylsulfate polyacrylamide gel electrophoresis (SDS-PAGE), and labeled using monoclonal primary Abs against anti-phospho-ZAP70 (Tyr319) (Cell Signaling, Beverly, MA, USA), anti-ZAP70 (Cell Signaling), anti-phospho-LAT (Tyr191) (Abcam, Cambridge, UK), anti-LAT (Thermo Scientific), anti-phospho-PLCγ1 (Tyr783) (Cell Signaling), anti-PLCγ1 (Abcam), and anti-β-actin (Abcam) Abs. The secondary Ab was labeled with HRP, and electrochemiluminescence was used to visualize the bands.

### Preparation of Lipid Raft Fractions

Enriched CD4^+^ T cells (2 × 10^7^) from C57BL/6 mice were stimulated for 24 h at 37°C with medium, an anti-CD3 Ab (1 µg/ml), an anti-CD28 Ab (1 µg/ml), and the TRAIL (10 µg/ml), or a combination of anti-CD3/anti-CD28 Abs and the TRAIL. Cells were subsequently lysed in 0.2% Triton-X, 50 mM Hepes, 100 mM NaCl, 5 mM EDTA, 1% Ser/Thr protein kinase inhibitor, 1% Tyr protein kinase inhibitor, and 1% protease inhibitor for 1 h on ice. The lysate was 1:1 diluted in 80% ice-cold sucrose in 150 mM NaCl, 5 mM EDTA, and 25 mM MES, transferred to Ultra-Clear centrifuge tubes (Beckman Coulter), and overlaid with ice-cold 30% sucrose, followed by ice-cold 5% sucrose. The sucrose gradients were centrifuged at 2 × 10^5^ *g* in a Beckman Coulter SW41 rotor for 22 h at 4°C. Twelve 375-µl fractions were collected and SDS-PAGE sample buffer was added to the harvested fractions, followed by a subsequent Western blot analysis.

### Intracellular Phospho-Protein Staining for Flow Cytometry

Measurement of phosphorylated protein by flow cytometry was processed as previously described (28). Briefly, cells were fixed with 4% paraformaldehyde for 45 min at RT, and permeabilized with ice-cold methanol for another 45 min at 4°C. The cells were then labeled with anti-phospho-ZAP70 Ab (Tyr319) (Cell Signaling, MA, USA) or anti-phospho-PLCγ1 Ab (Tyr783) (Cell Signaling, MA, USA) and analyzed by flow cytometry.

### Terminal Deoxynucleotidyl Transferase dUTP Nick End Labeling (TUNEL) Staining

To evaluate the apoptotic cell death within the spinal cords of EAE mice, three unstained sections corresponding to the HE-stained spinal cord slides were deparaffinized, and stained for apoptosis using DeadEnd™ Fluorometric TUNEL Assay (Promega, WI, USA) following the manufacturer’s instructions. The positive control slides were prepared by DNase I pretreatment of spinal cord slides from control mice before TUNEL staining. TUNEL-FITC^+^ cells indicate apoptotic cells.

### Generation of MOG_35–55_-Specific CD4^+^ T Cells and I-A^b^ MOG_35–55_ Tetramer Staining

C57BL/6 mice were immunized with the MOG_35–55_ peptide (200 µg/mouse, s.c.) in a CFA emulsion at the first day (Day 0). Twelve days after the immunization (Day 12), splenic CD4^+^ T cells were then isolated by using MACS beads (STEMCELL Technologies, Vancouver, Canada) and cultured in RPMI 1640 medium supplemented with 1% FBS and 25 µg/mL I-A^b^ MOG_35–55_ peptide (Medical and Biological Laboratories, Japan) at 37°C for 3 days. The cells were washed twice with PBS and then stained with I-A^b^ MOG_35-55_ tetramer-PE (10 μg/mL in final concentration) (Medical and Biological Laboratories, Japan) and CD4-APC-Cy7 (5 μg/mL in final concentration) (Biolegend, San Diego, CA, USA) for 1 h at 4°C in PBS containing FBS (2.5%) for analysis by flow cytometry. MOG_35–55_-specific CD4^+^ T cells were defined as I-A^b^ MOG_35–55_ tetramer^+^ CD4^+^ cells by flow cytometry analysis.

### Statistical Analysis

Experimental autoimmune encephalomyelitis clinical scores were analyzed by the two way ANOVA tests to estimate the association between the two mice groups. Non-parametric Mann–Whitney *U*-test was used for comparison of different parameters in T cell *in vitro* assays between the two mice groups. A *p*-value <0.05 was considered statistically significant. All analyses were conducted using SAS software, ver. 9.4 (NC, USA). For RNA seq, CummeRbund was employed to perform statistical analyses of gene expression profiles in each group for the differential expression analysis. The reference genome and gene annotations were retrieved from the Ensembl database. *p*-value of ≤0.01 were considered significant.

## Results

### TRAIL Suppresses Neuroinflammation and Autoreactive T Cell Reactivity in EAE Mice

**Figure 1:**
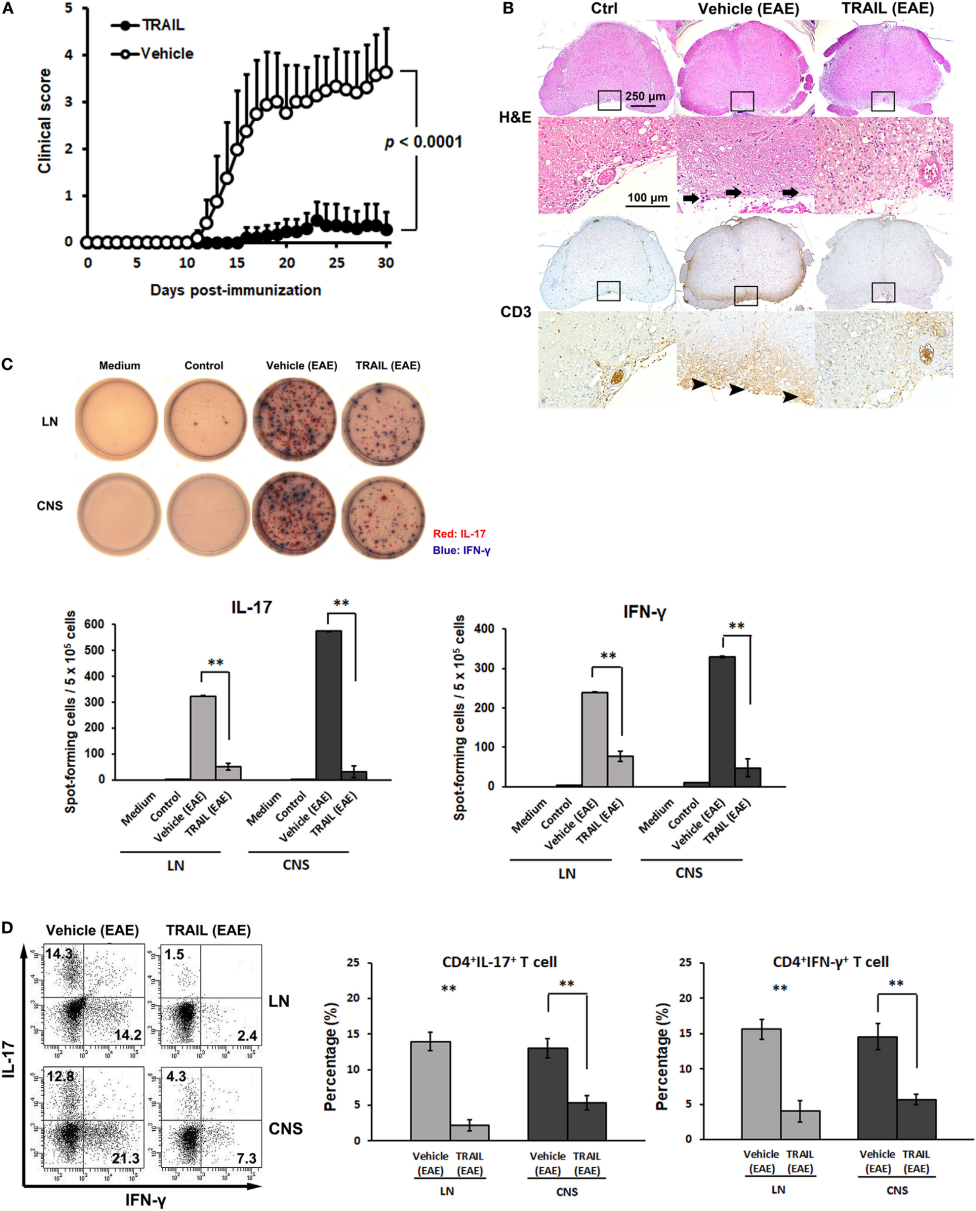
TRAIL suppressed neuroinflammation and T cell reactivity to neuroantigen in mice with experimental autoimmune encephalomyelitis (EAE). C57BL/6 mice were immunized by an s.c. injection with 200 µg the myelin oligodendrocyte glycoprotein (MOG)_35–55_ peptide emulsified in CFA containing 500 µg heat-killed *Mycobacterium tuberculosis* H37RA on day 0. Pertussis toxin, at 250 ng per mouse, was i.p. injected on the day of immunization (day 0) and 24 h later (day 1). From 48 h postimmunization (day 2), mice were treated with either vehicle (200 µl/mouse/day, i.p.) or TRAIL (100 µg/mouse/day, i.p.). **(A)** Mice were monitored daily for clinical paralysis, and the daily mean clinical score ± SD is presented for each group (*n* = 15 in each group). Statistics were calculated by the two way ANOVA test. **(B)** Spinal cords were isolated and examined histologically from control and EAE mice treated with the vehicle or TRAIL on day 30 after immunization. Cross-sections of paraffin-embedded spinal cords were stained with hematoxylin and eosin (H&E) and an anti-CD3 immunohistochemical antibody. Staining is representative of sections taken from five mice per group. Arrows indicate inflammatory cells. Arrow heads indicate CD3^+^ T cells. **(C)** Lymph node (LN) and central nervous system (CNS) mononuclear cells were isolated from control and EAE mice treated with vehicle or TRAIL on day 30 after immunization and restimulated with MOG_35–55_ (20 µg/ml) for 24 h. MOG_35–55_-activated interleukin (IL)-17 (red) and interferon (IFN)-γ (blue) responses were analyzed by a dual color ELISPOT assay. Representative figures of each group are shown (upper panel), and the frequency of MOG_35–55_-activated IL-17 and IFN-γ secretion was quantified as spot-forming cells per 5 × 10^5^ cells (lower panel). **p < 0.01 by non-parametric Mann–Whitney *U*-test. Data are representative of at least six independent experiments. **(D)** LN and CNS mononuclear cells were isolated from EAE mice treated with the vehicle or TRAIL on day 30 after immunization and restimulated with phorbol 12-myristate 13-acetate/ionomycin in the presence of GolgiSTOP for 5 h. IL-17 and IFN-γ production by LN and CNS CD4^+^ cells was measured by intracellular cytokine staining. Data are representative of three independent experiments, each using pools of three mice for each group (left panel). Percentages of CD4^+^ IL-17^+^ and CD4^+^IFN-γ^+^ cells were quantified (right panel). **p < 0.01 by non-parametric Mann–Whitney *U*-test.

To address the role of TRAIL in regulating autoimmune inflammation, TRAIL was administered to mice with induced EAE, a prototype for T cell-mediated autoimmune disease. As illustrated in Figure 1A, prompt neurological defects developed from day 12 in the control group after mice were induced with EAE, and the severity of disease activity increased in a time-dependent manner. In contrast, in EAE mice treated with TRAIL, only very mild neurological deficits developed over the entire 30-day experimental course. Furthermore, in the histopathological analysis, TRAIL significantly suppressed neuroinflammation and reduced T cell infiltration in spinal cords (Figure 1B). To further determine whether TRAIL affects the reactivity of encephalitogenic T cells, we investigated MOG_35–55_-specific T cell responses in EAE mice treated with TRAIL. As shown in Figure 1C, mononuclear cells isolated from LNs and the CNS of vehicle-treated EAE mice produced significant amounts of Th1- and Th17-type cytokines (i.e., IFN-γ and IL-17) in response to the MOG_35–55_ peptide. This was profoundly diminished in EAE mice treated with TRAIL. Furthermore, numbers of IL-17- and IFN-γ-producing T cells that had infiltrated into the CNS were greatly reduced in TRAIL-treated EAE mice compared to vehicle-treated EAE mice in flow cytometric analyses (Figure 1D). Taken together, our results indicate that TRAIL treatment significantly reduced disease severity and neuroinflammation in EAE mice, and T cell reactivity against neuroantigen was profoundly inhibited by TRAIL.

### Suppression of Neuroinflammation by TRAIL Is Dependent on TRAIL-R Signaling

**Figure 2:**
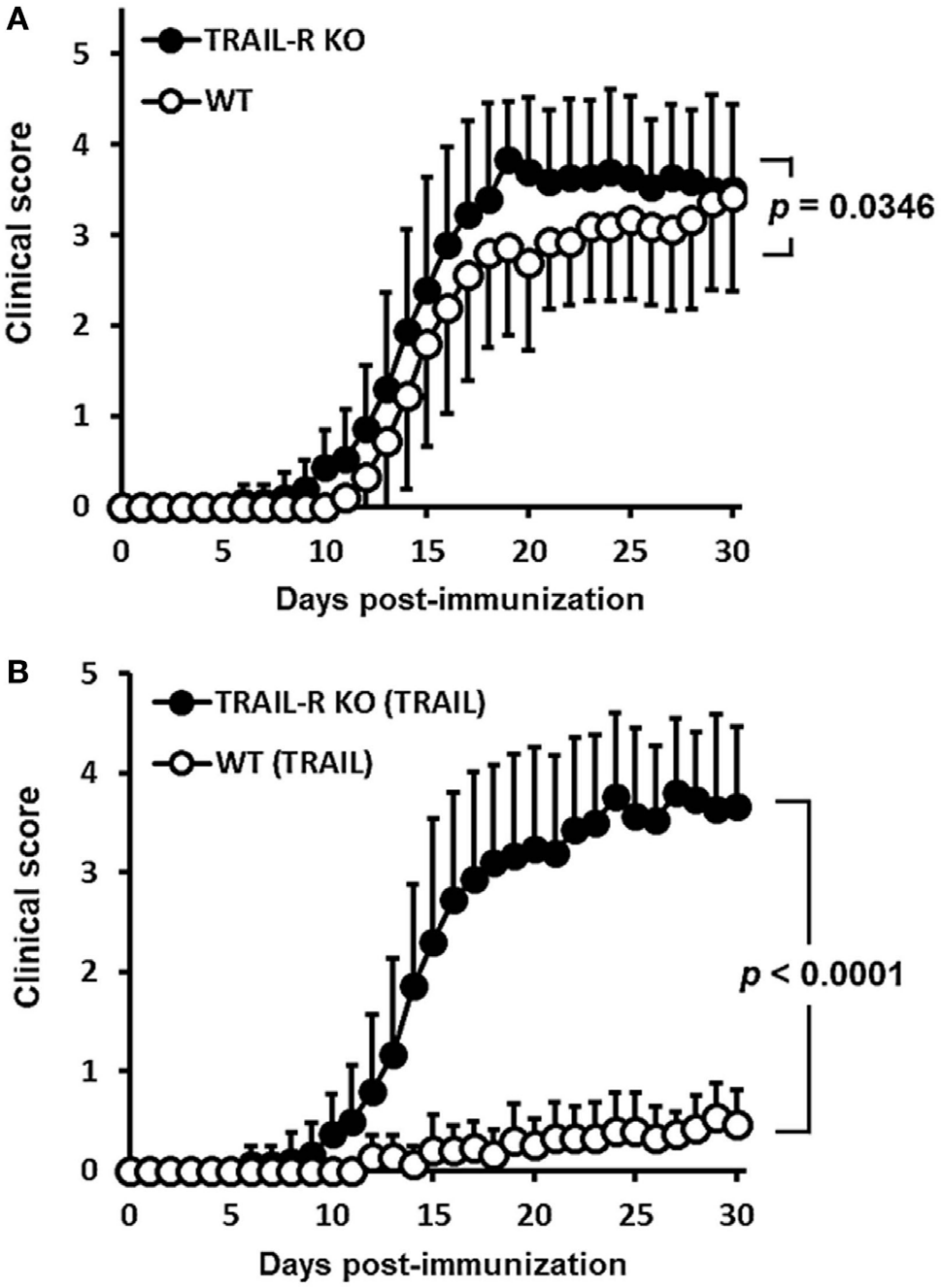
TRAIL-mediated inhibition of neuroinflammation in experimental autoimmune encephalomyelitis is through TRAIL-R. **(A)** Wild-type (WT) and TRAIL receptor-knockout (TRAIL-R KO) mice were immunized with the myelin oligodendrocyte glycoprotein (MOG)_35–55_ peptide (200 µg/mouse, s.c.) in a complete Freund’s adjuvant (CFA) emulsion on day 0 followed by pertussis toxin (250 ng/mouse, i.p.) on days 0 and 1. Clinical scores among groups were measured at the indicated time points (*n* = 15 in each group). Statistics were calculated by the two way ANOVA. **(B)** WT and TRAIL-R KO mice were immunized with the MOG_35–55_ peptide (200 µg/mouse, s.c.) in a CFA emulsion on day 0 followed by pertussis toxin (250 ng/mouse, i.p.) on days 0 and 1. Beginning on day 2 after immunization, mice were treated daily with TRAIL (100 µg/mouse, i.p.). Clinical scores among groups were measured at the indicated time points (*n* = 15 in each group). Statistics were calculated by the two way ANOVA.

To confirm that the suppression of neuroinflammation by TRAIL was dependent on TRAIL/TRAIL-R interaction, we further investigated TRAIL-induced anti-inflammatory effects in the EAE model using TRAIL-R KO mice. Results illustrated in Figure 2A demonstrated that more-rapid onset and enhanced disease severity score developed in TRAIL-R KO mice in which EAE was induced compared to those in WT mice. Moreover, when treated with TRAIL, the anti-inflammatory effects of TRAIL were abolished in TRAIL-R KO mice (Figure 2B). All these results indicate that TRAIL-mediated suppression of neuroinflammation in EAE is dependent on TRAIL-R signaling.

### TRAIL Directly Inhibits MOG_35–55_-Activated T Cell Activation and Suppresses the Development of Autoimmune Inflammation in Adoptive Transfer-Induced EAE

**Figure 3:**
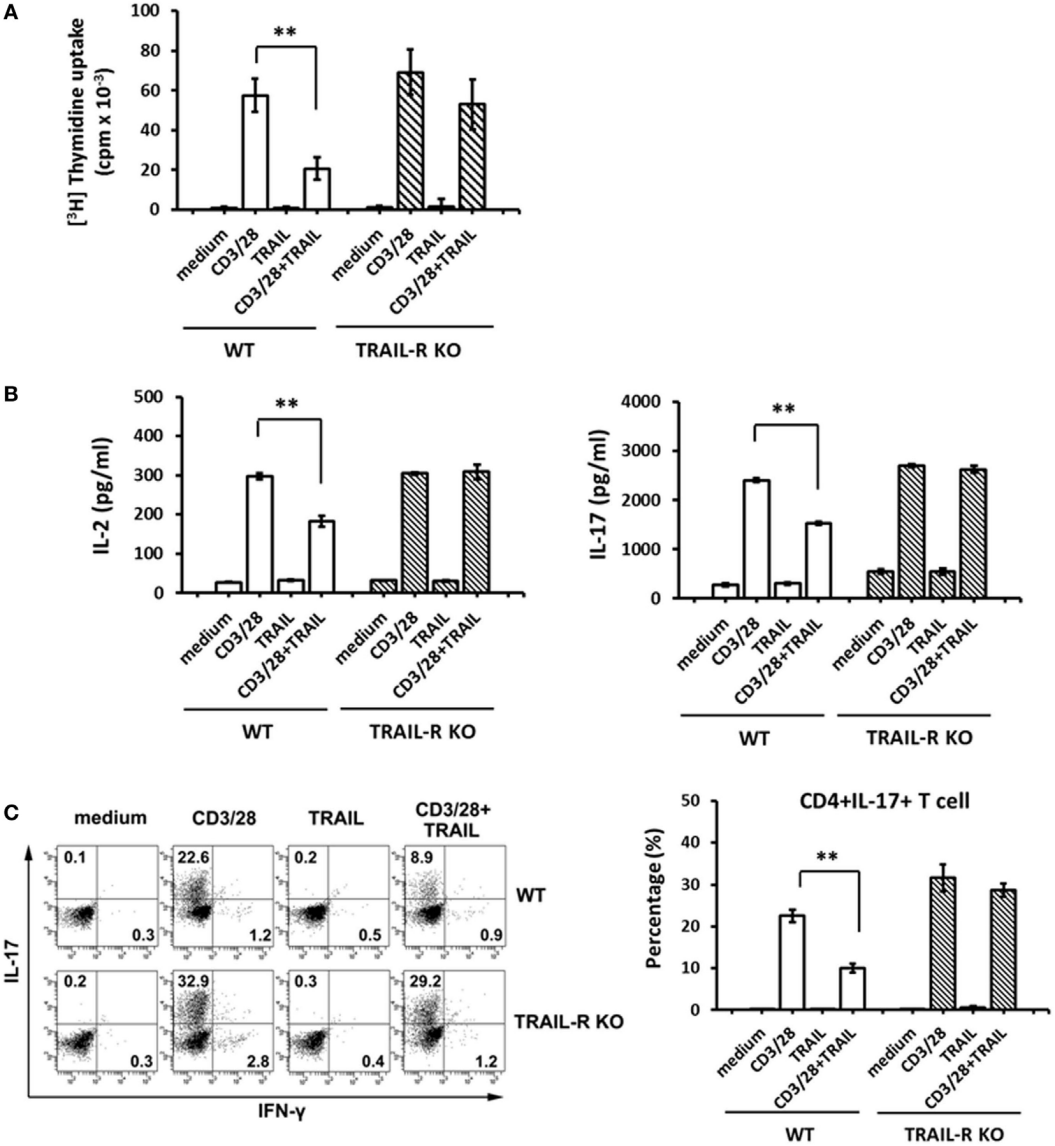
TRAIL inhibited myelin oligodendrocyte glycoprotein (MOG)_35–55_-activated T cells. Th17-polarized MOG_35–55_-activated splenic T cells were generated from wild-type (WT) and TRAIL-R KO mice after experimental autoimmune encephalomyelitis immunization, as described in Section “Materials and Methods.” **(A)** 10^5^ MOG_35–55_-activated splenic Th17 cells were cultured in 96-well flat-bottomed plates precoated with medium, an anti-CD3 antibody (Ab; 1 µg/ml), an anti-CD28 Ab (1 µg/ml), and TRAIL (10 µg/ml), or a combination of anti-CD3/anti-CD28 Abs and TRAIL for 96 h; then [^3^H] thymidine was added for an additional 16 h. Radioactivity (presented as cpm) was determined with a scintillation counter. **p < 0.01 by non-parametric Mann–Whitney *U*-test. **(B)** 2 × 10^5^ MOG_35–55_-activated splenic Th17 cells were cultured in 96-well flat-bottomed plates precoated with medium, anti-CD3/anti-CD28 Abs, and TRAIL, or a combination of anti-CD3/anti-CD28 Abs and TRAIL for 24 h. Supernatants were collected and assayed for interleukin (IL)-2 (left panel) and IL-17 (right panel) concentrations by an enzyme-linked immunosorbent assay. Data are shown as the mean ± SD of triplicate samples. **p < 0.01 by non-parametric Mann–Whitney *U*-test. **(C)** IL-17 and interferon (IFN)-γ production by MOG_35–55_-activated splenic Th17 cells were measured by intracellular cytokine staining following stimulation with medium, anti-CD3/anti-CD28 Abs, and TRAIL, or a combination of anti-CD3/anti-CD28 Abs and TRAIL for 24 h. Data are representative of three independent experiments (left panel), and percentages of CD4^+^ IL-17^+^ cells were quantified (right panel). **p < 0.01 by non-parametric Mann–Whitney *U*-test.

Accumulating evidence (14, 15) implies that TRAIL inhibits autoimmune inflammation *via* an apoptosis-independent pathway. Our recent report also demonstrated that TRAIL could inhibit T cell activation and suppress inflammatory arthritis in an apoptosis-independent manner (18). Similarly, in this study, EAE mice treated with TRAIL showed no increased apoptotic cells within spinal cord, and pan-caspase inhibitor failed to abolish the anti-inflammatory effect of TRAIL in EAE (Figure S1 in Supplementary Material). In addition, when T cells from EAE mice were re-stimulated with anti-CD3/CD28 in the presence or absence of TRAIL, there were no increased apoptotic cells compare to the cells without treatment (Figure S2 in Supplementary Material), indicating TRAIL inhibits T cell activation *via* a pathway distinct from inducing cell apoptosis in EAE. To further investigate whether TRAIL directly inhibits MOG_35–55_-responsive T cell activation and suppresses the development of autoimmune disease, we first examined the effects of TRAIL on MOG_35–55_-activated T cell proliferation and cytokine production. As shown in Figure 3, when MOG_35–55_-activated Th17 cells from WT mice were stimulated with anti-CD3/CD28 in the presence of TRAIL, T cell proliferation was significantly inhibited by TRAIL (Figure 3A). More importantly, this inhibitory effect of TRAIL was completely abolished in TRAIL-R KO mice. Furthermore, TRAIL directly inhibited IL-2 and IL-17 production (Figure 3B) as well as the generation of IL-17-positive cells (Figure 3C) in MOG_35–55_-activated Th17 cells. Similarly, these effects were also abrogated in TRAIL-R KO mice. Moreover, when I-A^b^ MOG_35–55_ tetramer^+^ CD4^+^ cells were restimulated with anti-CD3/CD28 in the presence or absence of TRAIL, there was no increased apoptotic cells among these MOG-specific T cells when compared to the cells treated with medium only, suggesting TRAIL did not trigger apoptosis in MOG-specific T cells or anti-CD3/CD28 activated T cells (Figure S3 in Supplementary Material). All these results indicate that TRAIL directly inhibits the activation and cytokine production by MOG_35–55_-activated Th17 cells.

**Figure 4:**
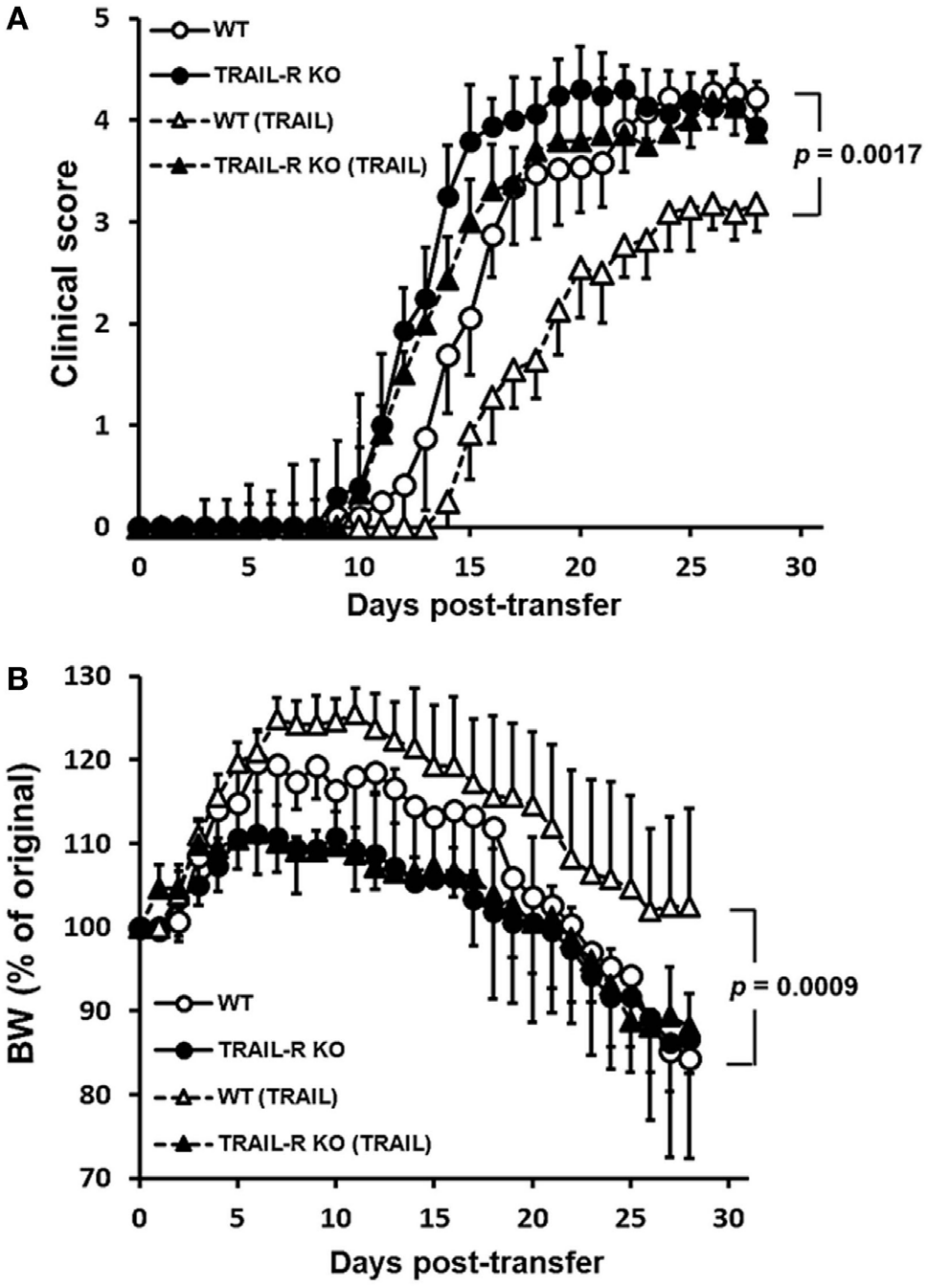
TRAIL-treated myelin oligodendrocyte glycoprotein (MOG)-activated T cells reduced disease activity in adoptive transfer-induced experimental autoimmune encephalomyelitis (EAE). Th17-polarized MOG_35–55_-activated T cells were generated from wild-type (WT) and TRAIL-R KO mice after EAE immunization, as described in Section “Materials and Methods.” MOG_35–55_-activated splenic Th17 cells were stimulated with anti-CD3 (1 µg/ml) and anti-CD28 (1 µg/ml) antibodies in the presence or absence of TRAIL (10 µg/ml) for 24 h and adoptively transferred into Rag1 KO mice (5 × 10^6^ CD4^+^ T cells/recipient). Adoptive transfer recipients were monitored daily for clinical paralysis and body weight changes. The daily **(A)** mean clinical score ± SD and **(B)** percentage of body weight change ± SD are presented for each group. Statistics were calculated by the two way ANOVA. Data shown are from one experiment with 12 mice/group representative of three performed.

To further evaluate whether TRAIL-treated MOG_35–55_-activated T cells suppress the development of autoimmune inflammatory disease, we adoptively transferred MOG_35–55_-activated splenic Th17 cells from WT and TRAIL-R KO mice into Rag1 KO mice to induce passive EAE. As shown in Figure 4A, after transfer of the activated T cells from WT mice, the recipient mice developed prompt neurological deficits from day 8 postadoptive transfer, and clinical scores increased over the entire 28-day experimental course. The onset of neurological deficits was earlier when the cells were transferred from TRAIL-R KO mice. In contrast, when TRAIL-treated MOG_35–55_-activated Th17 cells were adoptively transferred, recipient mice showed delayed onset and less-severe neurological deficits over the entire experimental course compare to those without TRAIL treatment. This effect was abolished when TRAIL-treated MOG_35–55_-activated Th17 cells from TRAIL-R-KO mice were adoptively transferred. Consistent with these results, recipient mice adoptively transferred with TRAIL-treated MOG_35–55_-activated Th17 cells from WT mice showed smaller but not significant body weight changes compared to the other adoptively transferred group (Figure 4B). To sum up, TRAIL directly inhibited T cell activation to neuro-antigen and thus suppressed the development of EAE.

### Downregulated TCR Signaling-Associated Genes in TRAIL-Treated EAE Mice

**Figure 5:**
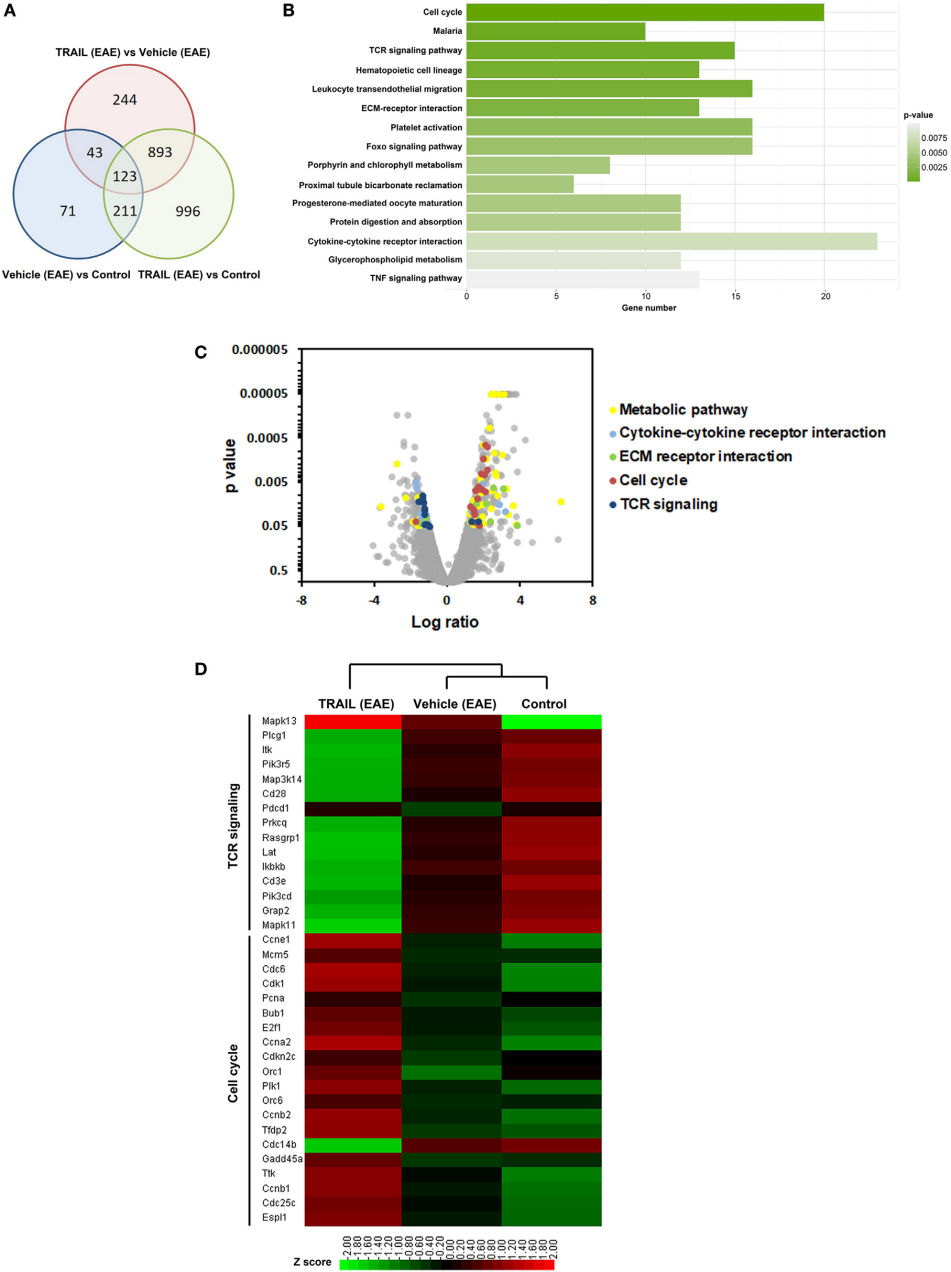
Downregulated T cell receptor (TCR) signaling-associated genes in TRAIL-treated mice with experimental autoimmune encephalomyelitis (EAE). C57BL/6 mice were immunized with the myelin oligodendrocyte glycoprotein_35–55_ peptide (200 µg/mouse, s.c.) in a complete Freund’s adjuvant emulsion on day 0 followed by pertussis toxin (250 ng/mouse, i.p.) on days 0 and 1. Beginning on day 2 after immunization, mice were treated daily with vehicle (200 µl/mouse/day, i.p.) or TRAIL (100 µg/mouse/day, i.p.). On day 15 after immunization, splenic CD4^+^ T cells were isolated from control and EAE mice after treatment with the vehicle or TRAIL. Total RNA of splenic CD4^+^ T cells from each group was extracted and sequenced on the Solexa platform (Illumina), as described in Section “Materials and Methods.” **(A)** Venn diagram showing the overlap among nominally significant genes of splenic CD4^+^ T cells from each group. **(B)** The number of significant genes in splenic CD4^+^ T cells from TRAIL-treated mice compared to vehicle-treated mice was plotted following a Kyoto Encyclopedia of Genes and Genomes (KEGG) pathway analysis. **(C)** Volcano plot showing distribution of up- and downregulated genes in splenic CD4^+^ T cells from TRAIL-treated mice compared to vehicle-treated mice following a KEGG pathway analysis. Significantly up- and downregulated genes are highlighted. **(D)** Heatmap representing gene expressions of splenic CD4^+^ T cells from each group. Only the most significant genes in TCR signaling and the cell cycle from the KEGG pathway analysis are shown. Statistical analysis was performed using R. Scale: Red indicates high expression and green is low expression.

To elucidate the potential immune-regulatory mechanism of TRAIL of activated T cells in EAE, we analyzed gene expression profiles of splenic CD4^+^ T cells from EAE mice treated with TRAIL by RNA sequencing and transcriptome analysis. We first checked the degree of overlap of differentially expressed genes among splenic CD4^+^ T cells from EAE mice treated with the vehicle or TRAIL and the control. As shown in Figure 5A, a number of significant genes overlapped among each group; however, there were still 244 genes that were significantly differentially expressed between splenic CD4^+^ T cells from TRAIL- and vehicle-treated EAE mice. Next, we analyzed these 244 significant genes categorized by a Kyoto Encyclopedia of Genes and Genomes (KEGG) pathway analysis. The results demonstrated that the most significantly enriched category for CD4^+^ T cells was “cell cycle” (multiple of enrichment: 3.13, *p* = 1.64 × 10^−5^) followed by “TCR signaling pathway” (multiple of enrichment: 2.80, *p* = 8.25 × 10^−4^) (Figure 5B). In addition, significant genes in the “TCR signaling pathway” tended to be downregulated while those in “cell cycle” tended to be upregulated in a volcano plot analysis (Figure 5C). In order to further analyze nominally significant genes in the “TCR signaling pathway” and “cell cycle,” we performed unsupervised hierarchical clustering (Figure 5D). Consistent with the volcano plot results, the heatmap showed that significant TCR signaling pathway-associated genes were downregulated, while significant cell cycle-associated genes were upregulated in CD4^+^ T cells from EAE mice treated with TRAIL. Altogether, these results showed that the gene transcription pattern from CD4^+^ T cells of TRAIL-treated EAE mice were involved in distinct TCR signaling and cell cycle pathways. Since TRAIL suppressed disease development, and these RNA seq results were in splenic CD4+ T cells from EAE mice treated with TRAIL or vehicle; it is still not able to exclude the possibility that the observed differences may be partly due to absence of disease (as an indirect effect of TRAIL) on the CD4+ T cells of the mice.

### TRAIL/TRAIL-R Interaction Suppresses Phosphorylation of Proximal TCR Signaling Molecules and Inhibits T Cell Activation

**Figure 6:**
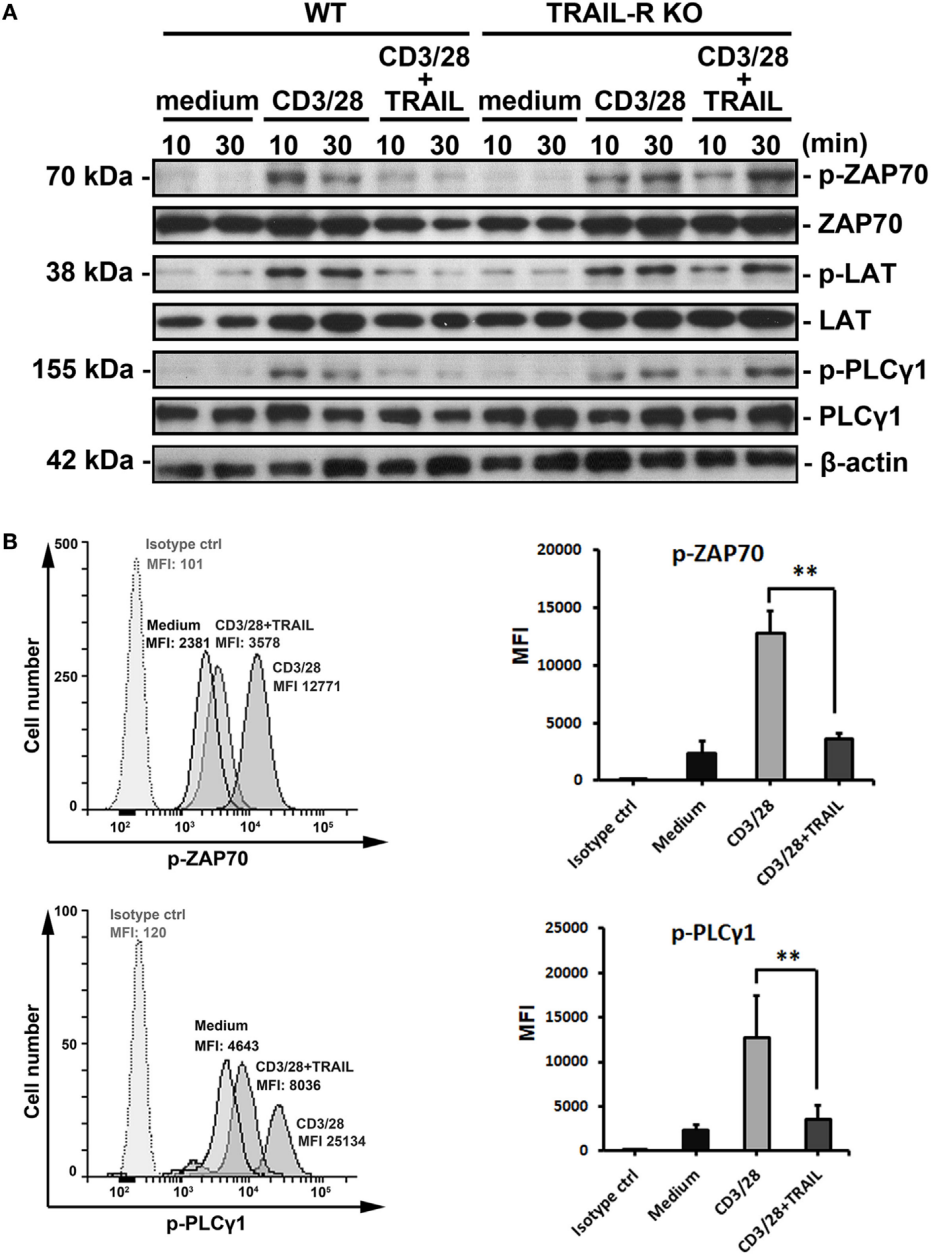

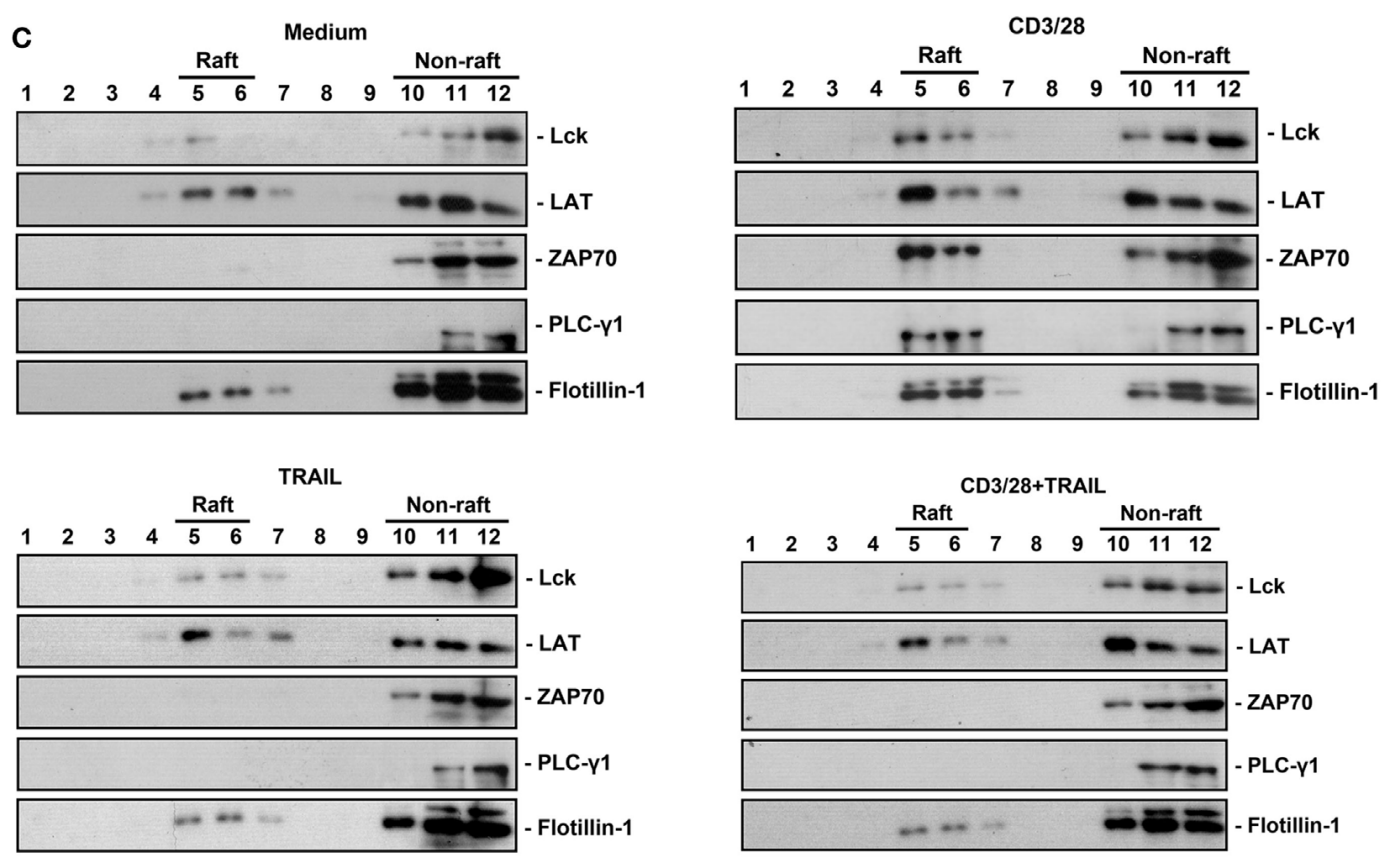
TRAIL inhibited T cell activation by suppressing phosphorylation of proximal T cell receptor signaling molecules. **(A)** 2 × 10^6^ primary splenic CD4^+^ T cells from C57BL/6 wild-type (WT) and TRAIL-R KO mice were stimulated at the indicated time point with medium, an anti-CD3 antibody (Ab; 1 µg/ml), an anti-CD28 Ab (1 µg/ml), and TRAIL (10 µg/ml), or a combination of anti-CD3/anti-CD28 Abs and TRAIL. Lysates of each group were prepared and immunoblotted with antiphospho-ZAP70 (Tyr319), anti-ZAP70, antiphospho-LAT (Tyr191), anti-LAT, antiphospho-PLCγ1 (Tyr783), anti-PLCγ1, and antiactin Abs. Blots are representative of three independent experiments performed. **(B)** 2 × 10^6^ primary splenic CD4^+^ T cells from C57BL/6 mice were stimulated for 2 h at 37°C with medium, anti-CD3/anti-CD28 Abs, TRAIL, or combination of anti-CD3/anti-CD28 Abs and TRAIL. Cells were fixed, permeabilized, and stained with anti-p-ZAP70 Ab or anti-p-PLCγ1 Ab. Representative figures for each group are shown (left panel) and quantified (right panel) from four independent experiments in each group. **p < 0.01 by non-parametric Mann–Whitney *U*-test. **(C)** 2 × 10^7^ primary splenic CD4^+^ T cells from C57BL/6 mice were stimulated for 24 h with medium, anti-CD3/anti-CD28 Abs, and TRAIL, or a combination of anti-CD3/anti-CD28 Abs and TRAIL. Cell were lysed and subjected to sucrose density gradient centrifugation to isolate lipid rafts. Proteins from equal volumes of representative collected fractions were immunoblotted with anti-Lck, anti-LAT, anti-ZAP70, anti-PLCγ1, and anti-flotillin-1 Abs. Blots are representative of three independent experiments performed.

To further confirm whether TRAIL directly inhibits activated CD4^+^ T cells and to investigate the possible mechanisms of TRAIL-mediated inhibition of T cell activation and regulation of the TCR signaling pathway, we isolated CD4^+^ T cells from WT and TRAIL-R KO mice and stimulated them with anti-CD3/ant-CD28 Abs in the presence or absence of TRAIL. As illustrated in Figure 6A, CD4^+^ T cells activated with anti-CD3/anti-CD28 significantly induced phosphorylation of proximal TCR signaling molecules, including ZAP70, LAT, and PLCγ1. In contrast, when CD4^+^ T cells were activated with anti-CD3/anti-CD28 Abs in the presence of TRAIL, phosphorylation of TCR-associated signaling molecules was profoundly inhibited. In addition, the TRAIL-mediated suppression of phosphorylated TCR-associated signaling molecules was abolished in TRAIL-R deficiency. Similarly, analysis of intracellular signaling by phospho-flow cytometry also illustrated that TRAIL inhibited phosphorylation of proximal TCR signaling kinases (Figure 6B; Figure S3 in Supplementary Material), indicating that TRAIL/TRAIL-R interaction inhibits T cell activation *via* interfering with phosphorylation of proximal TCR signaling molecules.

Because downstream TCR signaling requires recruitment of proximal TCR molecules into lipid rafts (29), we next analyzed whether TRAIL/TRAIL-R interaction also inhibited recruitment of these molecules into lipid raft microdomains. Lipid rafts are composed of flotillin-1 and constitutively express the adaptor protein, LAT. As shown in Figure 6C, when CD4^+^ T cells were activated by anti-CD3/anti-CD28 Abs, ZAP70, Lck, and PLCγ1 were recruited into lipid rafts. In contrast, recruitment of these proximal TCR signaling molecules into lipid rafts was abrogated in CD4^+^ T cells activated with anti-CD3/anti-CD28 Abs in the presence of TRAIL. Furthermore, confocal analysis also demonstrated that anti-CD3/anti-CD28 Ab-stimulated CD4^+^ T cells induced lipid raft assembly and recruitment of ZAP70, whereas these effects were abolished in the presence of TRAIL (Figure S4 in Supplementary Material), suggesting TRAIL directly inhibits proximal TCR signaling and interferes these molecules recruitment into lipid raft microdomain. Taken together, these results clearly indicate that TRAIL-R signaling directly inhibits phosphorylation of TCR-associated proximal tyrosine kinases and subsequent lipid raft recruitment, resulting in suppression of TCR downstream signaling and T cell activation.

## Discussion

In this study, we demonstrate that TRAIL directly inhibits T cell activation, resulting in suppression of autoimmune encephalomyelitis. Also, we revealed a novel immunoregulatory mechanism of TRAIL of inhibiting T cell activation through interrupting a proximal TCR signaling pathway. In addition, TRAIL exerts its anti-inflammatory effects directly through inhibiting T cell activation *via* TRAIL-R signaling, suggesting an apoptosis-independent pathway in suppressing inflammatory arthritis by TRAIL, and a novel role for TRAIL in regulating CD4^+^ T cell activation and modulating autoimmune diseases.

In recent years, accumulating evidences have demonstrated that TRAIL modulates immune responses in autoimmune diseases (14, 15, 19). Although mechanisms by which TRAIL inhibits inflammation in autoimmune diseases remain to be elucidated, most previous studies attributed the anti-inflammatory effects to the proapoptotic activity of TRAIL *via* triggering apoptosis in inflammatory cells (12, 22, 30, 31). Recent emerging studies suggested that the anti-inflammatory effects of TRAIL are not due to promoting cell apoptosis in several autoimmune animal models (13–15). In agreement with those observations, our recent study clearly demonstrated that the anti-inflammatory effect of TRAIL in autoimmune arthritis is *via* an apoptosis-independent pathway; importantly, TRAIL exerts its anti-inflammatory effects by directly inhibiting T cell activation *via* TRAIL/TRAIL-R interaction (18). Similar results can also be observed in EAE model (Figures S1 and S2 in Supplementary Material). All these results suggest a novel apoptosis-independent, immune-regulatory role for TRAIL in modulating autoimmune diseases, and raise the potential therapeutic implication of TRAIL in autoimmune diseases.

Experimental autoimmune encephalomyelitis, a T cell-mediated autoimmune disease, is a chronic and multiphasic autoimmune inflammatory disorder of the CNS, and is thought to be triggered by myelin-specific CD4^+^ Th1 and Th17 cells (32). Previous studies revealed a controversial role of TRAIL in EAE. TRAIL blockade in EAE mice (14) or TRAIL-deficient mice with EAE induction (21) were more prone to neurological deficits, and delivery of genetically modified dendritic cells expressing TRAIL prevented the development of EAE (22). Nevertheless, while a brain-specific blockade of TRAIL after EAE induction reduced the clinical severity, intracerebral delivery of TRAIL into EAE mice increased clinical deficits (31). All of those results indicate that TRAIL/TRAIL-R has a dual role as a regulator of immune cell function and as an effector of cytotoxicity during immune responses. In present study, we explicitly demonstrated that systemic administration of TRAIL inhibited the clinical severity and neuroinflammation in EAE. In addition, neuroantigen-specific T cell responses from EAE mice treated with TRAIL were profoundly suppressed, indicating that TRAIL serves as an inhibitory ligand in regulating T cell autoimmunity. Both TRAIL- and TRAIL-R-deficient mice were shown to be more susceptible to autoimmune induction (18, 21, 33, 34). In accordance with those observations, our results demonstrated that neurological deficits in TRAIL-R KO mice with EAE induction were exacerbated, and the inhibitory effect of TRAIL in autoimmune responses was dependent on TRAIL-R signaling. Hence, our results support the notion that TRAIL/TRAIL-R interaction regulates excessive inflammation and controls autoimmunity.

In the present study, we demonstrated that TRAIL directly inhibited MOG_35–55_-activated T cell proliferation and cytokine production and clearly defined that this effect was dependent on TRAIL-R signaling. Furthermore, TRAIL-treated MOG_35–55_-activated T cells failed to induce neurological deficits after being adoptively transferred into recipient mice. Some earlier *in vitro* studies reported that activated T cell responses could be suppressed by TRAIL (15, 35), but little is known about the mechanism though which TRAIL controls T cell autoimmunity. Our results provide *in vivo* evidence that TRAIL suppresses autoreactive T cells and prevents the development of autoimmune disease *via* TRAIL-R signaling through an apoptosis-independent pathway.

In this study, a vast number of genes of CD4^+^ T cells were regulated by TRAIL in EAE mice when genetic networks were analyzed. Importantly, most such genes were involved in the cell cycle, TCR signaling pathway, extracellular matrix receptor interactions, cytokine-cytokine receptor interactions, and metabolic pathways (Figure 5). Of these pathways, most cell cycle-associated genes were upregulated, while genes associated with the TCR signaling pathway were downregulated by TRAIL, indicating a different regulatory role of TRAIL in the T-cell cell cycle and TCR signaling. Despite this discrepancy, the overall effects of TRAIL on proliferation of activated CD4^+^ T cells were inhibitory, suggesting that the effects of downregulation of TCR signaling-associated genes dominated cell cycle-associated genes. Indeed, some earlier studies demonstrated that TRAIL can form a “secondary complex” that transduces a non-apoptosis prosurvival signal through activating the nuclear factor-κB and c-Jun N-terminal kinase pathways (36, 37). In addition, prolonged TRAIL exposure upregulated IFN pathway-related genes in a gene microarray analysis (38). Those studies may partially explain why CD4^+^ T cells from TRAIL-treated EAE mice exhibited upregulated cell cycle genes. In contrast, TRAIL directly downregulates TCR signaling pathway-associated genes, and it provides another possible mechanism for TRAIL inhibiting T cell responses. Furthermore, to confirm whether TRAIL could directly inhibit activated T cells, we demonstrated in this study that upon TCR stimulation, TRAIL-treated T cells inhibited phosphorylation of proximal TCR signaling-associated molecules, including ZAP70, Lck, LAT, and PLCγ1, with repressed recruitment of these signaling molecules into lipid rafts. All these results provide a novel mechanism of TRAIL-R signaling in suppressing T cell activation, a novel signaling pathway distinct from traditional death receptor signaling.

Death domain–containing receptors of TNF superfamily can induce apoptosis when ligating to corresponding ligands. Interestingly, a conserved phosphotyrosine-containing motif within the death domain of these receptors seems to mediate inhibitory functions in activated immune cells (39). The death domain of TRAIL-R also contains this conserved YxxL motif and can recruit SHP-1 upon stimulation of T cells with TRAIL treatment (Figure S5 in Supplementary Material). This may be one possible mechanism that TRAIL-R signaling can directly inhibit TCR signaling.

The involvement of regulatory T cells (Tregs) in negative regulation of immune responses has been addressed in many autoimmune diseases. Previous study has reported that adoptive transfer of Tregs could protect the recipient mice from MOG-induced EAE (40). In addition, Hirata et al. further reported there were increased Foxp3^+^ cells in the spinal cord of EAE mice after adoptive transfer of genetically modified dendritic cells expressing both MOG and TRAIL (41). These findings suggest that TRAIL may have dual role in regulation of proinflammatory T cells and Tregs.

Taken together, we provide a novel mechanism of TRAIL/TRAIL-R interaction regulating CD4^+^ T cell activation and suppressing proximal TCR signaling in T cell activation *via* an apoptosis-independent pathway in autoimmune inflammation. Our results suggest that TRAIL-R can serve as a novel immune checkpoint in T cell responses and sheds light on future therapeutic applications for targeting TRAIL/TRAIL-R in autoimmune inflammatory diseases.

## Ethics Statement

The management and experimental procedures with animal were approved by the Animal Ethics Committee of the National Taiwan University Medical Center.

## Author Contributions

I-TC, H-FT, and P-NH designed research; I-TC, H-FT, C-CS, and P-NH performed research; H-FT, C-SW, and P-NH analyzed data; I-TC, H-FT, C-SW, and P-NH wrote the manuscript.

## Conflict of Interest Statement

The authors declare that the research was conducted in the absence of any commercial or financial relationships that could be construed as a potential conflict of interest.
