# Supplementary material for: TRAIL-Mediated Suppression of T Cell Receptor Signaling Inhibits T Cell Activation and Inflammation in Experimental Autoimmune Encephalomyelitis
Source: Front Immunol. 2018 Jan 22;9:15. doi: 10.3389/fimmu.2018.00015 (PMC5786528; doi:10.3389/fimmu.2018.00015)
Supplement: Supplementary file 1 [file Presentation_1.PDF]

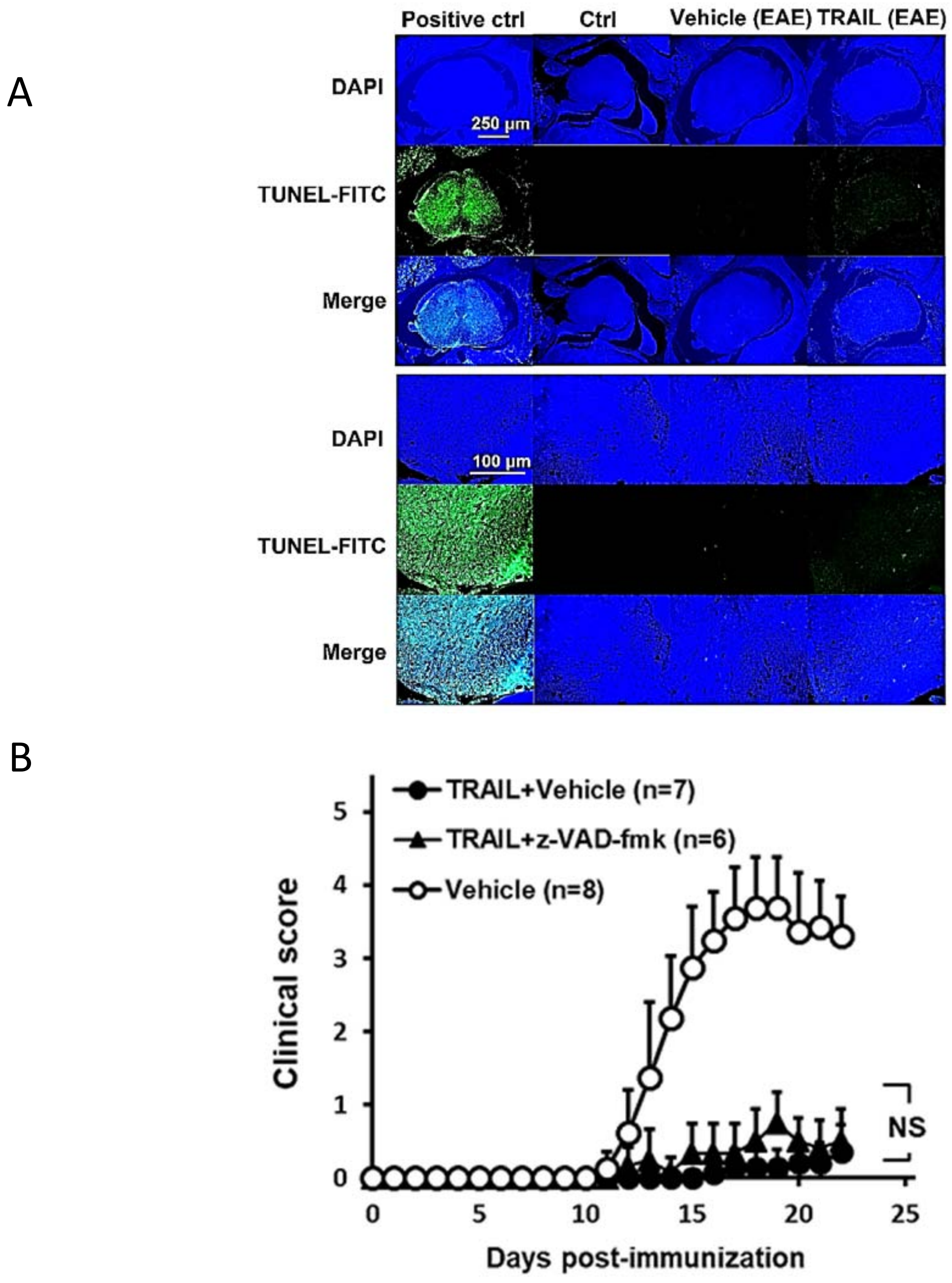

Fig. S1. Pan-caspase inhibitor failed to reverse TRAIL-mediated inhibition of neuroinflammation in EAE.

C57BL/6 mice were immunized by MOG $_{35\text{-}55}$ peptide emulsified in CFA on day 0. Pertussis toxin was injected on day 0 and day 2. (A) Mice were treated with either vehicle (200 μl/mouse/day, i.p.) or TRAIL (100 μg/mouse/day, i.p.) since day 2. Terminal deoxynucleotidyl transferase dUTP nick-end labeling (TUNEL) staining of the spinal cord in each group was carried out on day 30. Positive control were DNase I pre-treated spinal cord slides from control mice and TUNEL-FITC^{+} cells indicate apoptotic cells. (B) Mice were treated with the vehicle, TRAIL (100 μg/mouse/day, i.p.) or TRAIL plus z-VAD-fmk (20 μg/mouse/day, i.p.) since day 2. Clinical scores among the groups were measured at the indicated time points. Statistics were calculated by the two way ANOVA tests between TRAILplus vehicle and TRAILplus z-VAD-fmk group. NS, not significant.
