# Supplementary material for: TRAIL-Mediated Suppression of T Cell Receptor Signaling Inhibits T Cell Activation and Inflammation in Experimental Autoimmune Encephalomyelitis
Source: Front Immunol. 2018 Jan 22;9:15. doi: 10.3389/fimmu.2018.00015 (PMC5786528; doi:10.3389/fimmu.2018.00015)
Supplement: Supplementary file 2 [file Presentation_2.PDF]

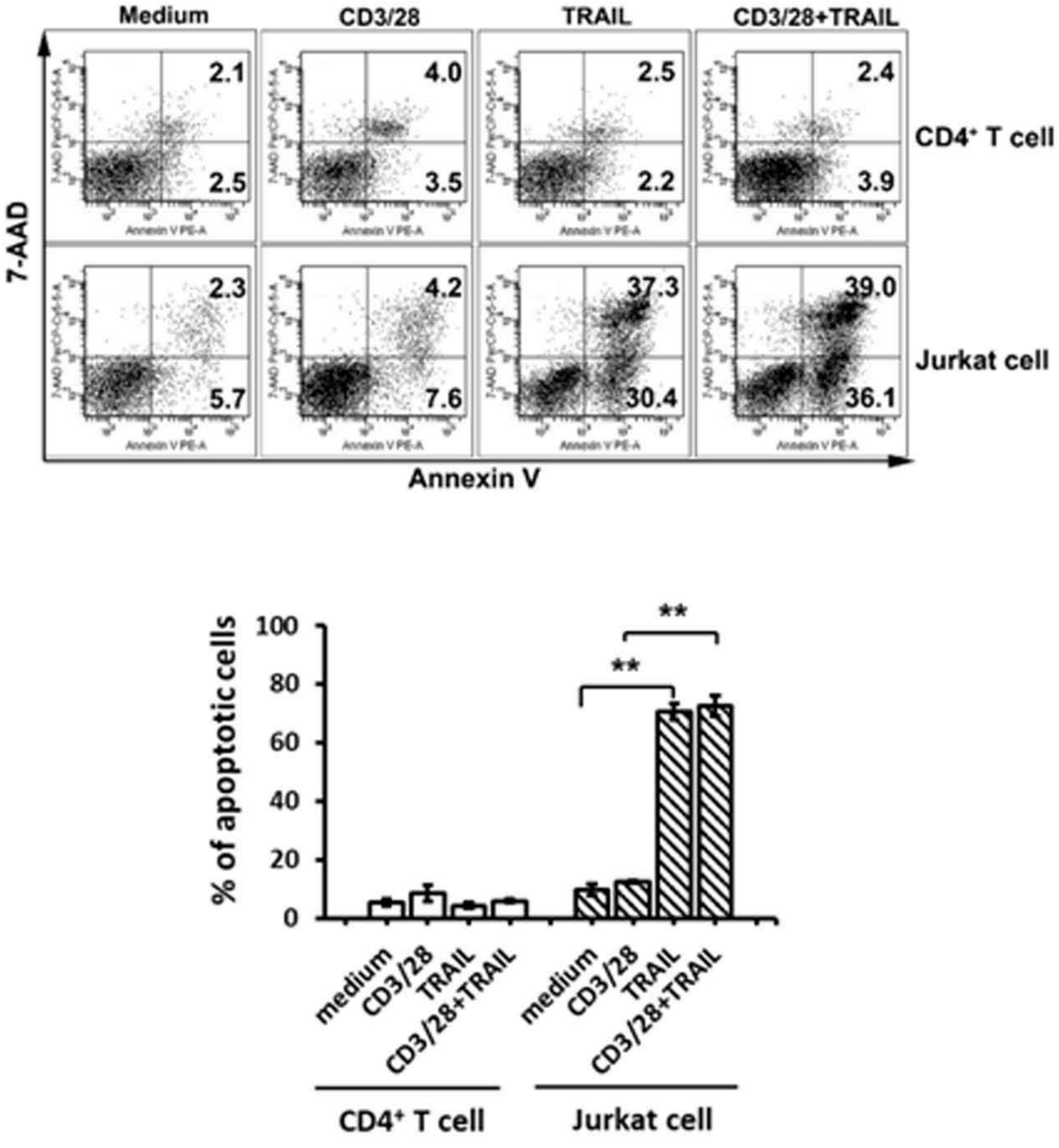

Figure S2. TRAIL did not promote cell apoptosis in activated T cell in EAE.
10^6 primary $CD4^+$ T cells from EAE mice and Jurkat cells were stimulated for 24 h with medium, anti-CD3/anti-CD28 Abs, TRAIL or combination of anti-CD3/anti-CD28 Abs and TRAIL, followed by Annexin V and 7-AAD staining. Representative figures of each group are shown (upper panel). The percentages of apoptotic cells, including Annexin V^+ 7-AAD^- (early apoptosis) and Annexin V^+ 7-AAD^+ (late apoptosis) cells, were quantified (lower panel). ** $P<0.01$ by non-parametric Mann-Whitney U test. The data are representative of at least three independent experiments in each group.
