# Supplementary material for: TRAIL-Mediated Suppression of T Cell Receptor Signaling Inhibits T Cell Activation and Inflammation in Experimental Autoimmune Encephalomyelitis
Source: Front Immunol. 2018 Jan 22;9:15. doi: 10.3389/fimmu.2018.00015 (PMC5786528; doi:10.3389/fimmu.2018.00015)
Supplement: Supplementary file 3 [file Presentation_3.PDF]

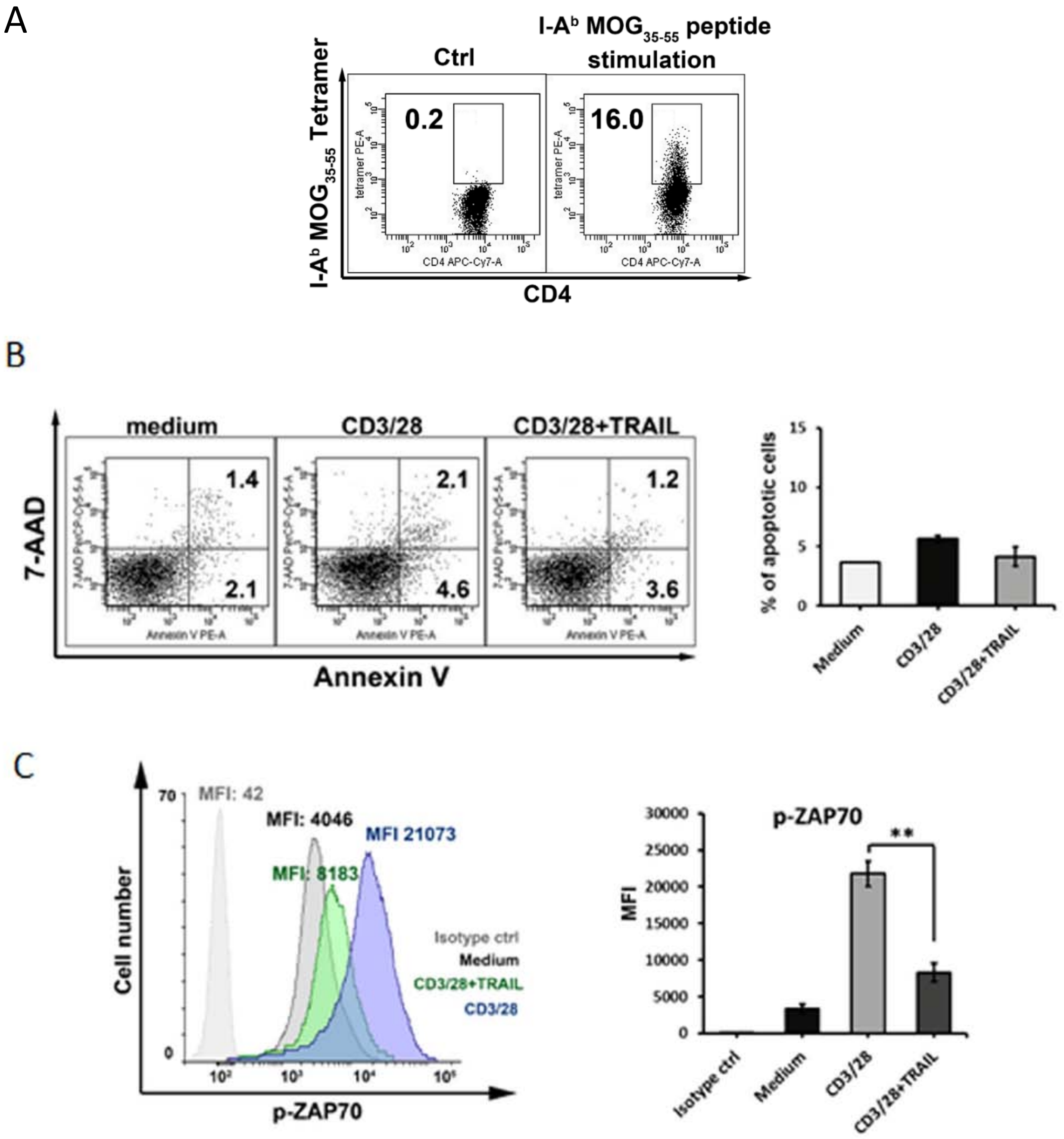

Figure S3. TRAIL inhibited phosphorylation of ZAP70 in activated MOG$_{35-55}$ -specific T cells without triggering apoptosis.

(A) C57BL/6 mice were immunized with the MOG$_{35-55}$ peptide (200 μg/mouse, s.c.) in a CFA emulsion for 12 days. CD4$^+$ T cells were isolated and restimulated with 25 μg/mL I-A^b MOG$_{35-55}$ peptide for 3 days then stained with MHC class II tetramer. I-A^b MOG$_{35-55}$ tetramer$^+$ CD4$^+$ cells were sorted and 2×10^5 cells were stimulated with medium, anti-CD3/anti-CD28 Abs or combination of anti-CD3/anti-CD28 Abs and TRAIL. (B) The cells were stained Annexin V and 7-AAD after 24 h stimulation and (C) anti-p-ZAP70 Ab after 1 h stimulation. Representative figures for each group ate shown and quantified. ** P<0.01 by non-parametric Mann-Whitney U test. The data are representative of triplicate experiments in each group.
