# Supplementary material for: TRAIL-Mediated Suppression of T Cell Receptor Signaling Inhibits T Cell Activation and Inflammation in Experimental Autoimmune Encephalomyelitis
Source: Front Immunol. 2018 Jan 22;9:15. doi: 10.3389/fimmu.2018.00015 (PMC5786528; doi:10.3389/fimmu.2018.00015)
Supplement: Supplementary file 4 [file Presentation_4.PDF]

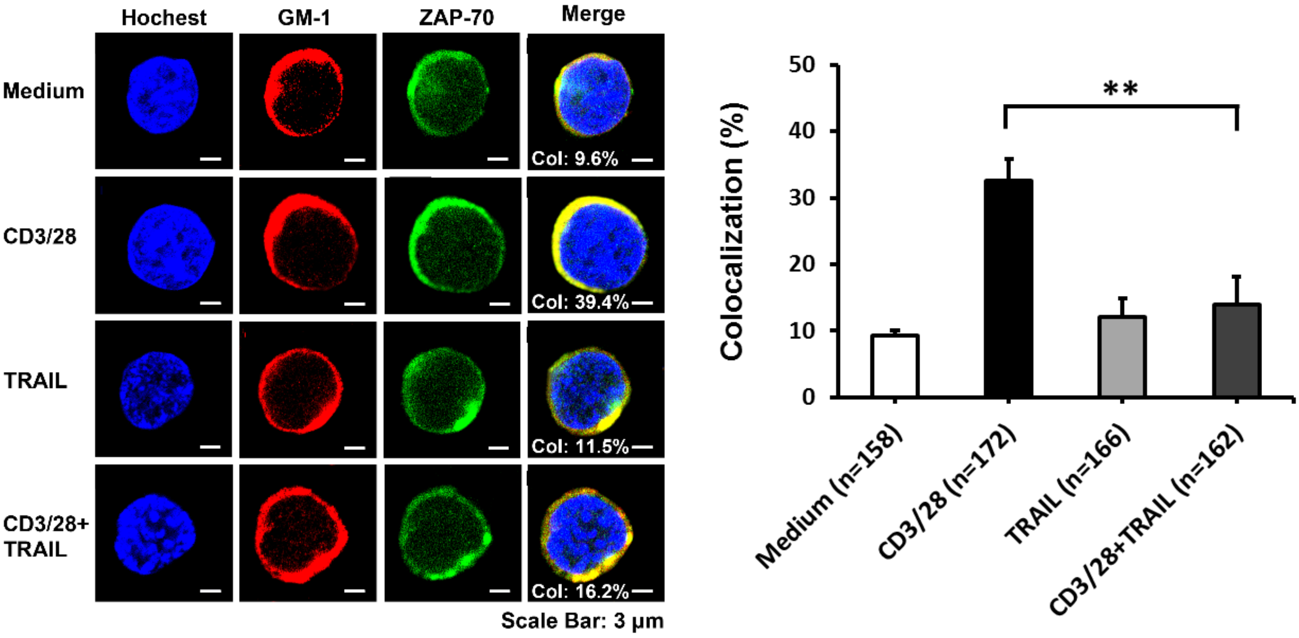

Figure S4. TRAIL inhibited recruitment of ZAP70 into lipid raft in activated T cells.
2×10^6 primary $CD4^+$ T cells from C57BL/6 mice were stimulated for 24 h with medium, anti-CD3/anti-CD28 Abs, TRAIL or combination of anti-CD3/anti-CD28 Abs and TRAIL. Cells were fixed, permeabilized, and processed for immunofluorescence staining of an anti-ZAP70 Ab (green), cholera toxin B subunit for GM1 (red), and Hoechst 33258 (blue). After staining, cells were plated and examined by Zeiss LSM-880 laser scanning confocal microscope (Carl Zeiss). Scale bars, 3 µm. Images for ratio of colocalization were processed and analyzed by ZEN 2 (Carl Zeiss). Data were means ± s.d. of triplicate samples. ** $p < 0.01$ by non-parametric Mann-Whitney U test.
