# Supplementary material for: TRAIL-Mediated Suppression of T Cell Receptor Signaling Inhibits T Cell Activation and Inflammation in Experimental Autoimmune Encephalomyelitis
Source: Front Immunol. 2018 Jan 22;9:15. doi: 10.3389/fimmu.2018.00015 (PMC5786528; doi:10.3389/fimmu.2018.00015)
Supplement: Supplementary file 5 [file Presentation_5.PDF]

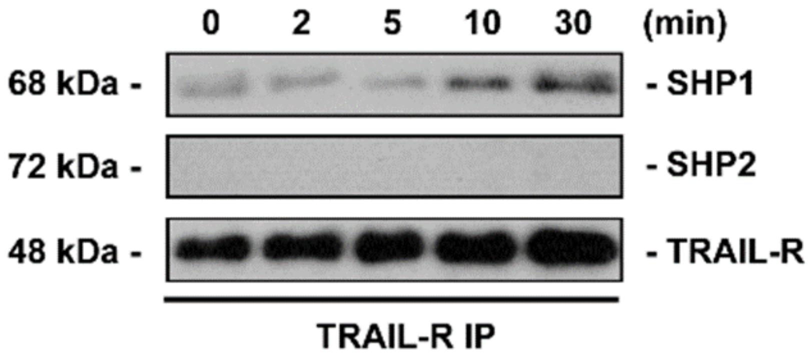

Figure S5. TRAIL induced recruitment of SHP1 to TRAIL-R.
5×10^6 primary $CD4^+$ T cells from C57BL/6 mice were stimulated with TRAIL (10 μg/ml) at indicated time point. Total cell lysates were immunoprecipitated with anti-TRAIL-R Ab, and then immunoblotted with anti-SHP1 and anti-SHP2 Abs.
